# Wnt signaling in development and disease

**DOI:** 10.1186/2045-3701-2-14

**Published:** 2012-04-20

**Authors:** Yingzi Yang

**Affiliations:** 1 Genetic Disease Research Branch, National Human Genome Research Institute, 49 Convent Drive, MSC 4472, Bethesda, MD, 20892, USA

## Abstract

Cell signaling mediated by morphogens is essential to coordinate growth and patterning, two key processes that govern the formation of a complex multi-cellular organism. During growth and patterning, cells are specified by both quantitative and directional information. While quantitative information regulates cell proliferation and differentiation, directional information is conveyed in the form of cell polarities instructed by local and global cues. Major morphogens like Wnts play critical roles in embryonic development and they are also important in maintaining tissue homeostasis. Abnormal regulation of these signaling events leads to a diverse array of devastating diseases including cancer. Wnts transduce their signals through several distinct pathways and they regulate vertebrate embryonic development by providing both quantitative and directional information. Here, taking the developing skeletal system as an example, we review our work on Wnt signaling pathways in various aspects of development. We focus particularly on our most recent findings that showed that in vertebrates, Wnt5a acts as a global cue to establishing planar cell polarity (PCP). Our work suggests that Wnt morphogens regulate development by integrating quantitative and directional information. Our work also provides important insights in disease like Robinow syndrome, brachydactyly type B1 (BDB1) and spina bifida, which can be caused by human mutations in the Wnt/PCP signaling pathway.

## Brief introduction of Wnt signaling pathways in development and disease

One of the most remarkable biological processes is the formation of a morphologically complex and functional diverse multicellular organism such as a human being from a single fertilized egg within a short period of time. During this tightly regulated process of embryonic morphogenesis, functional tissues and organs are formed and they have to be properly maintained during adult lives. Because cell-cell signaling plays essential and pivotal roles in both embryonic development and adult physiology, understanding the function and the underlying molecular mechanism of key cell signaling pathways in both development and diseases has been a major focus of our lab. Here we primarily focus on Wnt signaling in major developmental events of the skeletal system.

Wnts are evolutionarily conserved major regulatory factors in both development and disease. Wnt signaling is required in most embryonic developmental processes in both invertebrates and vertebrates. Abnormal Wnt signaling causes many types of tumors [1-3]. For instance, ectopic activation of *Wnt1* gene expression in the mouse mammary gland leads to tumor formation [4]. Mutations in Wnt signaling components have also been found to cause other human diseases. Weakened Wnt/β-catenin signaling leads to osteoporosis-pseudoglioma syndrome due to reduced bone mass [5] whereas enhanced Wnt/β-catenin signaling causes thick bone syndrome due to increased bone mass [6,7]. In addition, Robinow syndrome and Brachydactyly Type B1 that are characterized by shortened skeletal elements are caused by mutations in the Wnt/planar cell polarity pathway components [8-11].

**Figure 1:**
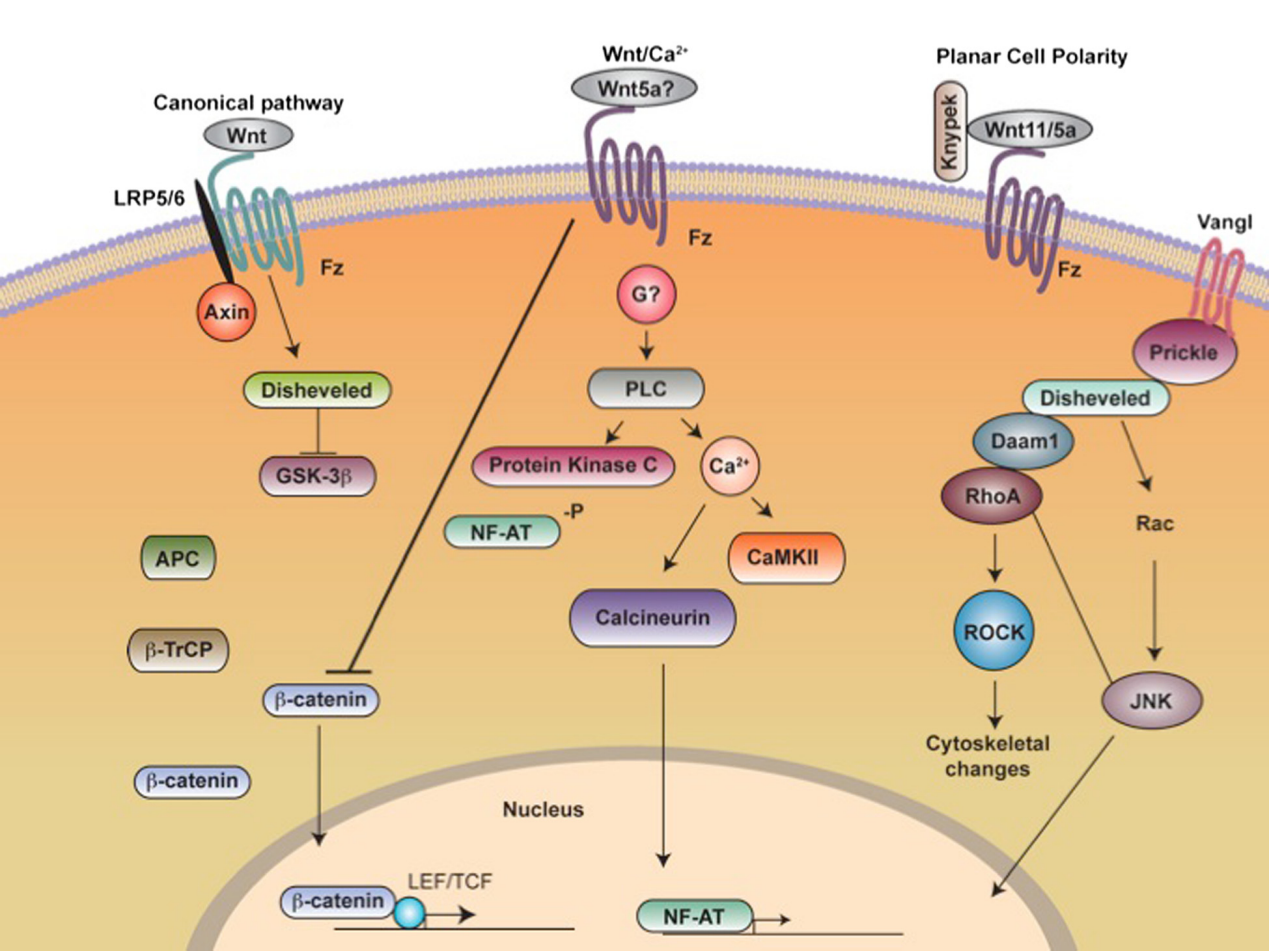
**Three representative Wnt signaling pathways. **See text for details.

Wnts are a large family of secreted molecules that can signal through several distinct pathways (Figure 1). The β-catenin mediated Wnt/β-catenin pathway is also called the canonical pathway. This pathway is best understood and mainly controls cell proliferation and differentiation. Central to this pathway is the control of β-catenin stability. In the absence of Wnt signaling, β-catenin is phosphorylated by GSK-3 in the “destruction complex” brought together by Axin and APC. Phosphorylated β-catenin was then recognized by the ubquitination machinery and sent to degradation in the proteosome. When Wnts bind to their receptors Frizzleds and Lrp5 or Lrp6, Lrp5/6 are phosphorylated and Dishevelled is activated, which lead to inactivation of the β-catenin “destruction complex” or disassembly of the β-catenin “destruction complex” such that β-catenin phosphorylation is reduced and stabilized. The stabilized β-catenin then translocates to the nucleus where it regulates downstream gene expression by binding to Lef/Tcf factors. Wnts may also signal through regulating intracellular Ca++ mobilization [12], but the regulation and functional significance of this pathway in mammalian development remain unclear. The planar cell polarity (PCP) pathway is also evolutionarily conserved and shares several components with the canonical pathway. Although Wnt ligands have not been found to regulate PCP in Drosophila [13], PCP is genetically regulated by *Wnt5a* and/or *Wnt11* in vertebrates [14-16]. Recent findings demonstrate that Wnt signaling acts through this pathway to provide directional information in controlling morphogenesis [17-20]. This part will be elaborated below.

## The skeleton provides a great system to study the functional mechanisms of Wnt signaling *in vivo*

The developing skeleton provides a good system to study major events in embryonic morphogenesis. It is relatively simple in its structure and cellular composition. It only contains three major cell types: chondrocytes that form the cartilage, osteoblasts that secrete bone matrix and osteoclasts that are specialized macrophage cells differentiated from hematopoietic precursor cells [21,22]. The skeletal system is widely distributed throughout the body, covering, supporting and protecting important organs. Compelling evidence also indicates that the skeletal system plays systemic regulatory roles as well [23]. The skeletal elements exhibit characteristic morphologies and organization that serve as convenient readouts for regulation of morphogenesis. For instance, the entire skeleton can be visualized in great detail in the intact embryo or adult animal after simple staining procedures.

Skeletal development starts from mesenchymal condensation in which mesenchymal progenitor cells are at least bipotential and they are also called osteochondral progenitors. These cells differentiate into either osteoblasts or chondrocytes depending on the mechanism of ossification. During intramembranous ossification that occurs primarily in the skull, osteochondral progenitors differentiate directly into bone forming osteoblasts whereas in endochondral ossification that occurs in most parts of the body, osteochondral progenitors first differentiate into cartilage forming chondrocytes instead. Osteoblast cells then form in the peripheral of the cartilage template and invade the hypertrophic cartilaginous area together with blood vessels to start the formation of trabecular bone. After bone formation, osteoclasts brought in by blood vessels remodels the bone, which is very important in maintaining bone homeostasis in adult lives [21]. The skeletal system is also highly segmented. Individual skeletal elements connect to each other through joints and this feature is required for motilities of vertebrate animals.

## The canonical Wnt signaling pathway controls cell fate determination in several fundamentally important processes of skeletal development

When my lab first got into the field of skeletal biology a decade ago, I decided to start by investigating the regulatory mechanism underlying several fundamentally important processes of skeletal development: the determination of chondrocytes versus osteoblasts when osteochondral progenitor cells are differentiating; The sequential proliferation and hypertrophy of chondrocytes in the long bone cartilage and induction of synovial joint formation. Now we found that it is quite remarkable that all these fundamental processes are controlled by Wnt/β-catenin signaling [17,24-30]. The functional spectrum of Wnt signaling in skeletal biology is getting broader and Wnt signaling has become a major field in skeletal biology.

**Figure 2:**
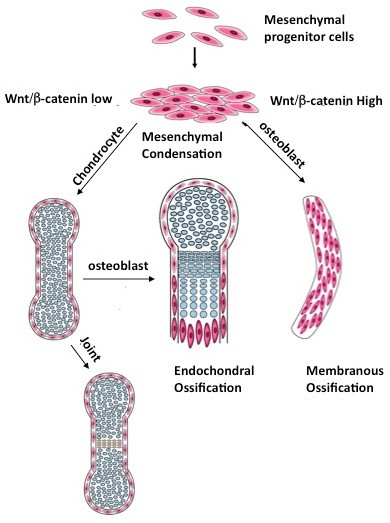
**Canonical Wnt signaling is both necessary and sufficient to induce synovial joint formation and determines osteoblast versus chondrocyte cell fates in skeletal development. **See text for details. This figure is adopted from [28,30].

To understand the functions of the canonical Wnt signaling in skeletal development, we utilized a canonical Wnt signaling reporter mouse strain *Top-Gal* in which *lacZ* expression reflects active canonical Wnt signaling. We found that in the *Top-Gal* mouse embryo, X-gal staining is selectively upregulated in the developing joint [28] marked by the expression of the earliest known joint marker *Gdf5*[31]. *LacZ* expression is also detected in the calvarium and perichchondrium where osteoblasts differentiate through intramembranous or endochondral ossification, respectively [30]. These results suggest canonical Wnt signaling may be important in joint formation and/or osteoblast differentiation regardless of ossification mechanisms [28,30]. Indeed we found by genetic manipulation of canonical Wnt signaling that this signaling pathway is both necessary and sufficient to induce joint formation [28] (Figure 2). The canonical Wnt pathway also controls bone and cartilage formation by controlling cell fate determination regardless of the ossification mechanism [30](Figure 2).

Taken together, we have found that the mesenchymal progenitor cells in the condensation are bipotential [28,30] (Figure 2). During intramembranous ossification, higher Wnt signaling in the condensation leads to inhibition of chondondrocyte differentiation and promotion of osteoblast differentiation. During endochondral ossification, however, Wnt signaling is kept low in the condensation such that only chondrocytes can differentiate. Later, when Wnt signaling is upregulated in the peripheral of the cartilage, osteoblasts will differentiate. Wnt signaling upregulation in the presumptive joint area induces joint formation. Therefore, by manipulating Wnt signaling, mesenchymal progenitor cells can be directed to form only chondrocytes or osteoblasts. These studies, together with others, have indicated that higher canonical Wnt signaling in cartilage may cause cartilage damages such as those observed in osteoarthritis [32]. Moreover, antibody therapies targeting canonical Wnt signaling are quite effective in increasing bone mass and are under clinical trials [33-36].

## The Wnt/PCP pathway controls skeletal morphogenesis by providing directional information

One of the most important functions of secreted molecules, Wnts included, is to act as morphogens which are critically required in embryonic morphogenesis by coordinating cell proliferation with cell fate determination [37-39]. This function has been the focal attention of developmental biology. However, the entire organism as well as the internal tissues and organs exhibit distinct morphologies and organizations which are essential for their functions. To understand how these are generated during morphogenesis, one has to understand how directional or polarity information is provided globally and locally. If cells in the developing embryo were not polarized and could not sense global or local directional cues, proliferation and differentiation would be the predominant mechanisms driving the development of the embryo. The result would be a three-dimensionally symmetric sphere composed of many different cell types. Therefore, providing directional information is fundamentally important in biology. For example, it is important to understand why the limb preferentially elongates along the proximal-distal axis.

**Figure 3:**
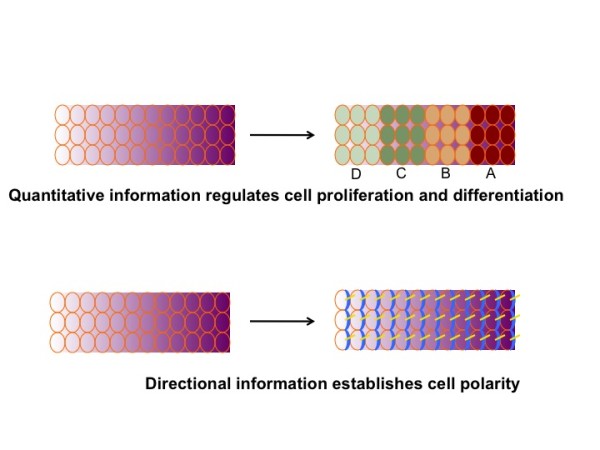
**Two fundamental roles of morphogen gradients in development. **It is well known that morphogen gradients provide quantitative information to coordinate proliferation with differentiation. For instance, morphogen gradients induce the production of different cell fates (A, B, C, D) according to thresh hold concentrations of the morphogen. Morphogen gradients may also integrate directional information by controlling planar cell polarity indicated by uniform asymmetrical localization of key polarity protein (shown in blue) and cellular behavior like the uniform orientation of cell extensions like cilia (yellow).

It is well understood that morphogens such as Wnts can form a concentration gradient across a field of hundreds of cells (Figure 3). This leads to the generation of different cell types in a distinct spatial order according to local threshold concentrations of the morphogen. In this regard, morphogen gradients provide quantitative information to generate a distinct pattern (Figure 3). For instance, the limb bud is patterned along the anterior-posterior and proximal-distal axes by morphogen gradients [40,41]. The fact that our limbs are elongated instead of being a three dimensionally symmetrical ball tells us that directional information has to be provided during pattern formation. However, it is unknown whether and how morphogen gradients also provide directional information (Figure 3). It is well known that a field of hundred of cells can acquire uniform planar cell polarity (PCP) which originally refers to the polarity in a plane perpendicular to the apical—basal axis [13,42-45]. PCP is evidenced by uniform asymmetrical localization of key polarity protein and uniform asymmetrical cellular behaviors such as the localization and orientation of cell extensions like cilia. Examples of PCP regulated processes are uniform orientations of Drosophila hair, mouse fur, inner ear sensory hair cells, elongating anteroposterior body axis and closing neural tubes. In a context dependent manner, the directional information provided by PCP is essential for cells to decide in which direction to divide, to move, or to interact with each other. As a class of critical morphogens, we thought Wnts may not jot just control cell proliferation and cell differentiation, they may also integrate the directional information by regulating PCP. But the underlying mechanism remained elusive. For many years it was not shown that morphogens can act as global cues to convey directional information via PCP [46,47]. Work from our laboratory was the first two show that secreted Wnts can establish directional information via the PCP pathway.

The PCP pathway is best characterized in Drosophila in which a group of core PCP components were identified and they play conserved roles in both invertebrates and vertebrates [13,42,43]. These core PCP components include Wnt receptor frizzled, a four transmembrane protein Van Gogh (vang) (the vertebrate homologues are Van Gogh like 1 and 2 (Vangl 1 and 2)), flamingo (the vertebrate homologues are Celsr1, 2 and 3), prickle and disheveled (Figure 1). Wnt ligands have not been found to regulate PCP in *Drosophila*. However, in vertebrates such the zebrafish, *Wnt11* and *Wnt5a* are both required to regulate PCP during convergent extension [48,49]. Downstream of the PCP core components, the PCP pathway might act through RhoA to control cytoskeleton remodeling and Jun kinase pathway to regulate gene expression, which still awaits rigorous genetic tests.

To investigate the function and mechanism of PCP, we removed both *Vangl1* and Vangl2 as they form the smallest family of core PCP components, so the problem of functional redundancy within a gene family can be managed relatively easily. In addition, unlike *Frizzled* and *DisheveledVangl1* and *2* are not shared by the canonical Wnt pathway. Therefore, the phenotypic perturbation in *Vangl1* and *2* mutants should be solely due to altered PCP. In mammals, PCP has been found to play fundamental roles in development. For instance, loss of PCP in the mouse *Vangl2 Looptail* (*Lp*) mutant leads to randomization of the inner ear hair cells and open neural tube [45,50-55]. Mild condition of incomplete neural tube closure called spina bifida. In humans, spina bifida is the most common neural tube defect and can be caused by mutations in both human VANGL1 and 2 [56,57]. Since the nature of the *Vangl2 Lp* mutation was not clear and the *Lp* homozygous mutant is embryonic lethal at the time, we generated null alleles of *Vangl1* and *Vangl2* and a floxed allele of *Vangl2*[51]. Both *Vangl1* and *2* regulates embryonic development as *Vangl1/2* double mutants are more severe than *Vangl2* the single mutant and *Vangl1* single mutant has no embryonic defects [51]. The various abnormalities demonstrated by the *Vangl1/2* double mutants indicate that PCP is fundamentally important in many developmental processes.

The earliest defect we can find in the *Vangl1/2* double mutant is in the establishment of left-right (L-R) asymmetry [51]. One of the molecular markers that allows one to detect left-right asymmetry in early embryos before any distinct morphological sign of left-right asymmetry shows up is unilateral expression of *Nodal* in the left lateral plate mesoderm of the early E8.5 mouse embryo [58,59]. In the *Vangl1/2* double mutant, *Nodal* expression is randomized indicating that L-R asymmetry is randomized and *Vangl1/2* mediated PCP acts upstream of *Nodal* expression to control L-R asymmetry.

The unilateral *Nodal* expression has to be triggered by an earlier event that breaks the bilateral symmetry. During vertebrate morphogenesis, establishment of L-R asymmetry follows the determination of dorsal-ventral (D-V) and anterior-posterior (A-P) body axes during gastrulation. In mice, bilateral symmetry is broken by a leftward fluid flow across a pit-like, teardrop-shaped node generated by posteriorly localized motile cilia of the node cells [60-63]. Both genetic analysis and mathematical modeling have shown that such leftward nodal flow is both necessary and sufficient to trigger unilateral *Nodal* expression. Abnormal cilium localization will result in turbulent nodal flow, which disrupts left-right patterning. Therefore, failure to transmit the A-P positional information in the node cells is likely to cause random cilium positioning and randomized L-R patterning. Thus, a mechanism that enables the node cells to interpret A-P positional information to position the nodal cilia sits at the top of the regulatory hierarchy of L-R asymmetry.

**Figure 4:**
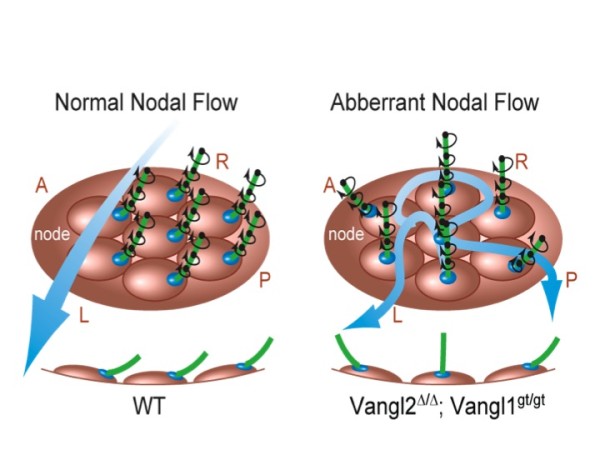
**PCP breaks bilateral symmetry during left-right patterning by positioning the nodal cilia to the posterior side of the node cells. **See text for details. This figure is adopted from (Song et al., 2010).

We found that the node cells are polarized by PCP along the A-P axis [51] (Figure 4). Both Vangl1 and Vangl2 protein were found to be selectively upregulated and asymmetrically localized in the forming node cells along the A-P axis. Outside of the node or prior to node formation, Vangl1 and Vangl2 protein levels were much lower and they were not asymmetrically localized. To test the functional significance of Vangl1/2 in node cell polarization, ciliogenesis and cilium localization were examined. In contrast to some previously published results in other systems, stringent genetic tests clearly show that PCP is not required for ciliogenesis [51,54]. However, in the Vangl or other PCP mouse mutants, cilia are randomly distributed around the center of the node cells, although Nodal cilia are mostly posteriorly localized in wild type embryos [51,54] (Figure 4). When the nodal flow is examined by following the paths of beads applied to the live embryos through live imaging, it was further discovered that randomization of nodal cilium localization leads to turbulent nodal flow [51,53] (Figure 4). In the control node, the bead paths were smooth and parallel to each other indicating the nodal flow is unidirectional. In the mutant, the bead paths contained abrupt turns, knots and often crossed with each other demonstrating the nodal flow is turbulent. Therefore, PCP is required to break the bilateral symmetry by establishing a unidirectional Nodal flow (Figure 4).

We then asked what establishes PCP in the developing embryo and whether PCP also regulates other important directional morphogenetic events such as limb elongation. Because the limb of the *Vangl* double mutant embryo is much shorter and distal digits were lost, we hypothesized that PCP controls directional limb elongation. Because *Wnt5a* is expressed in a graded manner in the early developing limb and primitive streak adjacent to the node and *Wnt5a*^*-/-*^ mutant embryos exhibits much shortened limb [25,64], we also hypothesized Wnt5a is a cue that establishes PCP in the embryo. Indeed, *Wnt5a* genetically interacts with *Vangl2* in neural tube closure and hair cell orientation in inner ear [16].

We then asked whether Wnt5a directs P-D limb elongation of the developing limb by regulating PCP. In the wild type limb, cartilage formation extends distally. But in the *Wnt5a*^*-/-*^ distal limb, cartilage failed to extend distally, forming ball-like structure [17,64]. We found previously that loss of distal digits in the *Wnt5a*^*-/-*^ embryo may be caused by upregulation of the anticondrogenic canonical Wnt signaling activity [25]. The ball-like structure of the forming digit cartilage led us to hypothesize that *Wnt5a* may also control cartilage elongation along the PD axis in limb development by regulating PCP.

To test whether the early differentiated chondrocytes are indeed polarized and show PCP, we examined Vangl2 protein localization and found that Vangl2 was asymmetrically localized along the P-D axis only in the Sox9-positive chondrocytes, not in the Sox9-negative interdigital mesenchymal cells in the limb [17]. These results demonstrate for the first time with a definitive molecular marker that chondrocytes are indeed polarized by PCP. Importantly, in the *Wnt5a*^*-/-*^ mutant limb, such polarized Vangl2 localization disappeared indicating that *Wnt5a* controls chondocyte polarity through PCP [17].

As *Wnt5a* genetically interacts with *Vangl2* and Wnt5a has been found to bind Ror2, a receptor tyrosine kinase [16,65], we further hypothesized that Wnt5a transduces its signal through a novel receptor complex containing Vangl2 and Ror2. Mutations in both WNT5A and ROR2 are found to cause Robinow syndrome characterized by shortened limb dwarfism [8-11]. To test this hypothesis further, we generated *Ror2* and *Vangl2* double mutant embryos and found that they phenocopied the *Wnt5a*^*-/-*^ embryo in the limb, craniofacial processes and the tail. The limb phenotypes of the Wnt5a mutants and the Ror2/Vangl2 double mutants are almost identical [17]. In addition, we found in the developing limb bud, Vangl2 antibodies can pull down Ror2. Furthermore, Vangl2 and Ror2 association is significantly enhanced by Wnt5a [17].

Then we asked how Wnt5a gradient leads to establishment of PCP. In another word, how the cells in the limbs sense the Wnt5a morphogen gradient and interpret the directional information [17]. We found that Wnt5a and many other Wnt ligands, when coexpressed with Ror2, enhanced Vangl2 phosphorylation. Expression of *Wnt5a* or *Ror2* alone also enhanced Vangl2 phorsphorylation to a lesser extent. The smear gel mobility shift pattern suggests that there are hyperphosphorylated and hypophosphrylated Vangl2, indicating that Vangl2 can be phosphorylated on multiple sites. Vangl2 phosphorylation is also detected *in vivo* in mouse tissues including the limb and brain [17]. Phosphorylation is abolished by calf intestinal phosphatase (CIP) treatment. In addition, Wnt5a -induced Vangl2 phosphorylation is much reduced in *Ror2*^*-/-*^ MEF cells, indicating that Wnt5a induces Vangl2 phosphorylation through Ror2 [17].

To further understand how Vangl2 phosphorylation is regulated by Wnt5a/Ror2 signaling, we took a great effort to map the phosphorylation sites of Vangl2 [17]. We found that phosphorylation of Vangl2 occurs on conserved Serine (Ser) and threonine (Thr) residues that are organized in two different clusters in the protein. We also demonstrated that phosphorylation within both clusters occurs in a progressive manner such that founder residues are phosphorylated before others [17]. The progressive nature of Vangl2 phosphoylation suggests that different levels of Vangl2 phosphorylation is regulated by distinct Wnt5a dosages and this may be an underlying mechanism whereby responding cells interpret Wnt5a dosages. Because in the developing limb bud, there is a *Wnt5a* expression gradient from distal to proximal sides, we tested this *in vivo* first in the limb bud. We dissected the limb bud into distal parts with higher *Wnt5a* expression, the middle parts with medium *Wnt5a* expression and proximal parts with lower *Wnt5a* expression. Indeed, we found that there is a progressive reduction of Vangl2 phosphoryation distal to proximal limb bud [17]. Importantly, in the *Ror2*^*-/-*^ and *Wnt5a*^*-/-*^ distal limb bud, hyperphosphorylated forms of Vangl2 were replaced by hypophosphorylated forms that are observed in proximal parts of limb bud. To further test that different levels of Vangl2 phosphorylation are induced by distinct dosages of Wnt5a, cells expressing *Vangl2* and *Ror2* were cocultured with various numbers of *Wnt5a*-expressing cells. Indeed, increasing Wnt5a dosages led to progressively more extensive Vangl2 phosphorylation [17]. These *in vivo* and *in vitro* studies demonstrate that regulating levels of Vangl2 phosphorylation is a way to sense the Wnt5a morphogen gradient.

**Figure 5:**
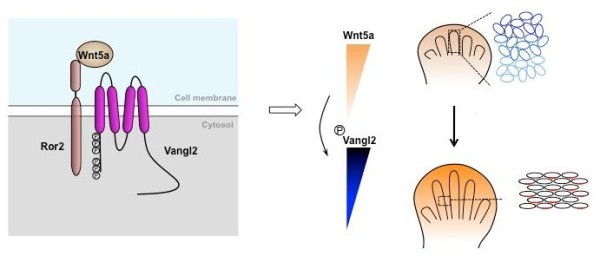
**Model of Wnt5a signaling gradient in establishing planar cell polarity. **See text for details.

We then tested whether phosphorylation regulates Vangl2 activity. We took advantage of the zebrafish *trilobite* mutants that are caused by *Vangl2* null mutations and exhibit convergent extension (CE) defects [49]. Wild type mouse *Vangl2* can rescue CE defects in 57% of the injected mutant fish embryos, a mutant vangl2 with S84A mutation (a founder site) rescued 31% of inject mutant embryos, whereas all phosphomutant had no rescuing ability. A Vangl2 phospho-mimicking mutant showed increased rescuing ability. Therefore, phosphorylation is required for Vangl2 activity and higher levels of Vangl2 phosphorylation led to higher Vangl2 activities. In the developing limb bud, by inducing different levels of Vangl2 phosphorylation, Wnt5a gradient is translated into an activity gradient of Vangl2 (Figure 5). The limb chondrocytes can orient themselves by sensing an initial small difference in Vangl2 activities in its immediate neighbors. This difference is then amplified by cell-cell interactions and positive feedback loops such that Vangl2 protein aggregates only on one side of the plasma membrane, laying the ground for further asymmetric cellular behavior, such as directional cartilage formation and growth.

Apart from its critical role in controlling PCP, our lab also showed that Wnt5a can promote chondrocyte differentiation by inhibiting the canonical Wnt signaling activity [25]. Therefore, Wnt5a controls both cell proliferation and polarity by coordinating canonical Wnt signaling with Wnt/PCP signaling. It is possible that these two Wnt pathways are mutually inhibitory to each other [66]. In the case of skeletal development, it will be interesting to test whether altered bone morphology due to disrupted PCP signaling also led to abnormal bone mass caused by alteration of canonical Wnt signaling.

## Misc

This review is organized largely based on my young investigator award lecture at the SCBA biannual meeting in Guang Zhou, 2011.

## Competing interests

The author declares that she has no competing interests.
